# Editorial: Solving the plasmalogen puzzle—From basic science to clinical application

**DOI:** 10.3389/fcell.2023.1137868

**Published:** 2023-01-16

**Authors:** Fabian Dorninger, Johannes Berger, Masanori Honsho

**Affiliations:** ^1^ Department of Pathobiology of the Nervous System, Center for Brain Research, Medical University of Vienna, Vienna, Austria; ^2^ Department of Neuroinflammation and Brain Fatigue Science, Graduate School of Medical Sciences, Kyushu University, Fukuoka, Japan; ^3^ Institute of Rheological Functions of Food-Kyushu University Collaboration Program, Kyushu University, Fukuoka, Japan

**Keywords:** plasmalogen, ether lipid, membrane, Alzheimer’s disease, phospholipid

In the almost 100 years since their discovery, numerous scientific breakthroughs around plasmalogens have been made, but at the same time the enigmatic phospholipid group is still keeping scientists of various disciplines on their toes. For example, biophysicists have revealed many biophysical properties of plasmalogens, but several aspects of their role in connection with other phospholipids in membrane bilayers remain cloudy. Geneticists have mostly elucidated the genetic basis of ether lipid biosynthesis but some genes involved in plasmalogen metabolism are still unknown; molecular biologists have identified new roles of plasmalogens in cellular processes but many puzzle pieces of the overall picture, particularly in complex organisms, have yet to be added; and clinicians have made major progress in diagnosing diseases involving plasmalogen deficiency but still lack suitable treatment strategies. Certainly, none of these scientific fields works independently, but an interdisciplinary approach is required to explore complex subjects like the biological role of plasmalogens. Many of the disciplines mentioned above have cooperated for the generation of the present Research Topic, which contains a balanced collection of reviews and original articles comprising a broad range of topics associated with plasmalogens, from basic science to clinical application.

Due to their unique biochemical structure and the particular challenge to discriminate the vinyl ether bond at the *sn*-1 position from double bonds of the alkyl chains of other ether lipid species, the reliable analytical determination of plasmalogens has bothered researchers for a long time (Koch et al., 2020). In their review article, Koch et al. give a comprehensive overview over the historic milestones in the analytical detection and quantification of plasmalogens. Subsequently, they highlight the progress made in recent years and demonstrate, how state-of-the-art methodology can be utilized to gather novel information on ether lipid species, their tissue distribution and physiological functions. In addition to technical advances in plasmalogen analysis, recent research has also brought key developments in elucidating the genes and enzymes involved in the generation of these lipids. One of the last orphan enzymes to be assigned to the corresponding gene was plasmanylethanolamine desaturase, the crucial enzyme for the introduction of the vinyl ether bond, which was recently shown by three independent approaches to be encoded by the *TMEM189* gene in humans (Gallego-Garcia et al., 2019; Werner et al., 2020; Wainberg et al., 2021). In the present Research Topic, Padmanabhan et al. outline the hunt for that gene and narrate, how the use of a comparatively unconventional model organism, *Myxococcus xanthus*, enabled the identification of this so crucial gene for plasmalogen biosynthesis. A more general overview of the steps involved in plasmalogen generation and their regulation is provided in the article authored by Dorninger et al. Moreover, the authors elaborate on the most recent knowledge on plasmalogen metabolism in different tissues as well as under physiological or pathological conditions and discuss the therapeutic potential of exogenous plasmalogen supplementation. While that article focuses mainly on plasmalogen metabolism in mammals, the work of Goldfine reminds us of the unusual distribution of plasmalogens in bacteria. As a long-standing expert in the topic (Goldfine, 2010), the author summarizes current knowledge on plasmalogen biosynthesis under anaerobic conditions and the functions of these lipids in bacterial species.

As a result of continuous research in the last years, important roles of plasmalogens in a variety of cell types and organs have emerged. Compared with other tissues, relatively little is known about their function in the eye. However, here, following up on the previous work of their group (Saab et al., 2014), Karadayi et al. reveal that plasmalogens are essential for the stability and functioning of gap junctions between Müller cells, a glia cell type specific for the retina, thus expanding our understanding of the role of plasmalogens in intercellular communication. On the cellular level, their involvement in signaling processes has become a major focus in the discussion around the physiological role of plasmalogens (Dorninger et al., 2020) and, accordingly, is also well represented in the current Research Topic. Honsho et al. have previously shown that plasmalogen levels in the inner leaflet of the plasma membrane bilayer govern a feedback loop that regulates the stability of FAR1, the rate-limiting enzyme in the plasmalogen biosynthesis pathway (Honsho et al., 2017). In their Original Research article in this Research Topic, they extend their findings and identify ATP8B2 as the flippase enzyme responsible for the asymmetric distribution of plasmalogens in the plasma membrane, thus controlling the feedback loop and, consequently, downstream processes like intracellular signaling. Previous work has shown that in the mammalian nervous system, plasmalogens are crucial for the adequate functioning of the Akt-ERK axis (da Silva et al., 2014), one of the main signaling cascades in many cell types, and that G-protein coupled receptors are important mediators of this effect (Hossain et al., 2016). Following up on these findings, Hossain et al. provide experimental evidence that signaling *via* brain-derived neurotrophic factor is impacted by plasmalogens as a downstream effect of the Akt-ERK modulation. Furthermore, they indicate that this mechanism is responsible for learning and memory deficits observed in mice upon plasmalogen deficiency, which can be alleviated by dietary plasmalogen supplementation.

Based on their association with such important processes like learning and memory, plasmalogens (or their deficiency) have been ascribed major contributions to various human diseases. The most obvious example is the inborn defect in plasmalogen biosynthesis, which causes the rare disorder rhizomelic chondrodysplasia punctata (RCDP). In this Frontiers Research Topic, Fallatah et al. use a series of mouse models with graded plasmalogen deficiency to show that genotype severity determines the levels of several biochemical markers. This renders these mouse models particularly valuable for the clinical evaluation of RCDP, where similar observations have been made (Fallatah et al., 2021). Excitingly, also neurochemical markers and the characteristic neurobehavioral alterations correlate with the degree of plasmalogen deficiency, thus paving the way for establishing novel readouts in clinical trials for this so far uncurable disease. Next to inborn errors in their biosynthesis, a role of plasmalogens has been implicated in a variety of other human diseases. One so far unknown example is studied by Karadayi et al. in the present Research Topic. The research group has previously shown that in children developing retinopathy of prematurity—an eye disease that can lead to early blindness and is associated with abnormal retinal vessel development—arachidonic acid in erythrocyte membranes accumulates with time *in utero* at the expense of docosahexaenoic acid (Pallot et al., 2019). Here, the authors utilize data from a prospective cohort study to support the hypothesis that plasmalogens play a major role in modulating the arachidonic acid/docosahexaenoic acid ratio in children with retinopathy of prematurity.

A disease that has kept the world in suspense in the last 3 years is COVID-19 caused by infection with the SARS-CoV-2 virus. Their previous work on lipids in sepsis (Amunugama et al., 2021) and the comparable disease courses in sepsis and severe COVID-19 prompted the authors around Pike et al. to study plasmalogen levels in a mouse model of SARS-CoV-2 infection. Remarkably, their experiments reveal a reduction of lung plasmalogen levels in the infected mice, thus showing similarities to the situation upon sepsis, for which the authors demonstrate a depletion of plasmalogens in plasma of both rats and humans. More than in any other disease, an involvement of plasmalogens is debated in the pathogenesis of Alzheimer’s disease (Senanayake and Goodenowe, 2019), the most common cause of dementia worldwide. Goodenowe and Senanayake add to the intrigue with a post-mortem analysis of temporal brain cortex samples from patients with mild cognitive impairment and Alzheimer’s disease compared with controls. The resulting data show an association with cognition and, under certain circumstances, even predictive value of specific ethanolamine phospholipid species, among them plasmalogens.

Their widespread involvement in disease mechanisms and maybe also etiology has raised speculation and curiosity about the potential of plasmalogens (or their precursors) as dietary supplements and therapeutic substances (Paul et al., 2019; Bozelli and Epand, 2021). A prerequisite for the evaluation of dietary supplementation strategies is an understanding of the metabolization of plasmalogens after their ingestion. Interestingly, previous work from Nishimukai and coworkers has shown that the extent of lymphatic absorption is heavily dependent on the type of headgroup (Nishimukai et al., 2011). In their article in the current Research Topic, Sato et al. investigate the metabolic fate of bacterial plasmalogens, which carry different *sn*-1 alkyl chains than those synthesized by mammals. Adding an important piece of knowledge on plasmalogen metabolism, they show that also these atypical plasmalogens are readily absorbed into the lymph after duodenal infusion and remodeled at the *sn*-2 position, similar to what is known for endogenous plasmalogen species. Several promising therapeutic approaches involving plasmalogens or enhancements thereof are featured in our Research Topic: Based on their previous expertise in evaluating potential drugs against Alzheimer’s disease (Chowdhury et al., 2021), Gu et al. utilize sea squirts to extract plasmalogens for the treatment of aged mice. Remarkably, intragastric application of the plasmalogen extract improved cognitive performance and aging-related molecular changes, thus raising hope for a future use as anti-neurodegenerative therapy. An alternative strategy is the use of precursor substances, which can be converted to plasmalogens *in situ* after oral application. One example is PPI-1011, a compound with a preformed vinyl ether bond at the *sn*-1 position, docosahexaenoic acid at *sn*-2 and lipoic acid as a stabilizer at *sn*-3 (Wood et al., 2011). Now, Smith et al. investigate in detail the metabolization and excretion of PPI-1011 in mice by using a radioactively labeled variant. The authors show the distribution of the label across various tissues, including the brain, which is an essential target for all therapeutic strategies in plasmalogen deficiency. In view of these data, we are eagerly awaiting future studies involving functional readouts after the supplementation with PPI-1011. An additional step is taken by Goodenowe et al., who have previously stressed the positive association between blood plasmalogens and cognition in humans (Goodenowe and Senanayake, 2019). Here, they orally provided a plasmalogen precursor and show increased serum plasmalogen levels, reduced signs of oxidative stress and even clinical relevance for cognitive function in a small sample of cognitively impaired trial participants, which warrants further investigation of this compound. Also Fujino et al. have previously emphasized the value of plasmalogens for cognition in certain subgroups of cognitively impaired patients (Fujino et al., 2017). In this Research Topic, they indicate surprisingly positive effects of plasmalogens in a very different population: Results after dietary supplementation of plasmalogens extracted from scallops suggested improved mental health and reduced fatigue in male students. Even though the molecular mechanisms underlying the beneficial effect of plasmalogens or their precursors on cognitive parameters remain largely unclear, these data further position these compounds as complementary therapeutic substances in various neurological diseases. From a different perspective, this is also underlined in the review article of Rong et al.: After previously pointing out potential anti-viral properties of plasmalogens (Deng and Angelova, 2021), the authors here analyze structural similarities between plasmalogens and platelet-activating factor (PAF), an ether lipid without vinyl ether bond. Based on these, they hypothesize that plasmalogens could fine-tune PAF-associated signaling, thus exerting anti-inflammatory function, which may be favorable in pathological conditions involving a pro-inflammatory milieu.

Overall, the variety of articles and wide range of subjects in the present Research Topic well reflect the variegated research landscape in the plasmalogen field and underline the versatility of this lipid subgroup. They also demonstrate that major progress is made continuously to understand the role of plasmalogens in biological systems, and exploit their therapeutic potential. However, as researchers we are well familiar with the fact that every solved scientific question raises several new ones and that is no different for the field of plasmalogens. Accordingly, the research around plasmalogens will remain vivid also in the future and we are looking forward to the next revelations around this fascinating group of phospholipids.
